# Influence of Orbital Character on the Ground State Electronic Properties in the van Der Waals Transition Metal Iodides VI_3_ and CrI_3_

**DOI:** 10.1021/acs.nanolett.2c01922

**Published:** 2022-08-30

**Authors:** Alessandro De Vita, Thao Thi Phuong Nguyen, Roberto Sant, Gian Marco Pierantozzi, Danila Amoroso, Chiara Bigi, Vincent Polewczyk, Giovanni Vinai, Loi T. Nguyen, Tai Kong, Jun Fujii, Ivana Vobornik, Nicholas B. Brookes, Giorgio Rossi, Robert J. Cava, Federico Mazzola, Kunihiko Yamauchi, Silvia Picozzi, Giancarlo Panaccione

**Affiliations:** † Laboratorio TASC, in Area Science Park, Istituto Officina dei Materiali (IOM)-CNR, S.S.14, Km 163.5, I-34149 Trieste, Italy; ‡ Dipartimento di Fisica, Università di Milano, Via Celoria 16, I-20133 Milano, Italy; § Institute of Scientific and Industrial Research, Osaka University, 8-1 Mihogaoka Ibaraki, Osaka 567-0047, Japan; ∥ Department of Precision Engineering, Graduate School of Engineering, Osaka University, 2-1 Yamadaoka, Suita, Osaka 565-0871, Japan; ⊥ ESRF, The European Synchrotron, 71 Avenue des Martyrs, F-38043 Grenoble, France; # Consiglio Nazionale delle Ricerche (CNR-SPIN), Unità di Ricerca presso Terzi c/o Università “G. D’Annunzio”, 66100 Chieti, Italy; ∇ NanoMat/Q-mat/CESAM, Université de Liège, B−4000 Liege, Belgium; ○ School of Physics and Astronomy, University of St. Andrews, St. Andrews KY16 9SS, United Kingdom; ◆ Department of Chemistry, Princeton University, Princeton, New Jersey 08540, United States

**Keywords:** Electronic structure, van der Waals systems, ARPES, DFT

## Abstract

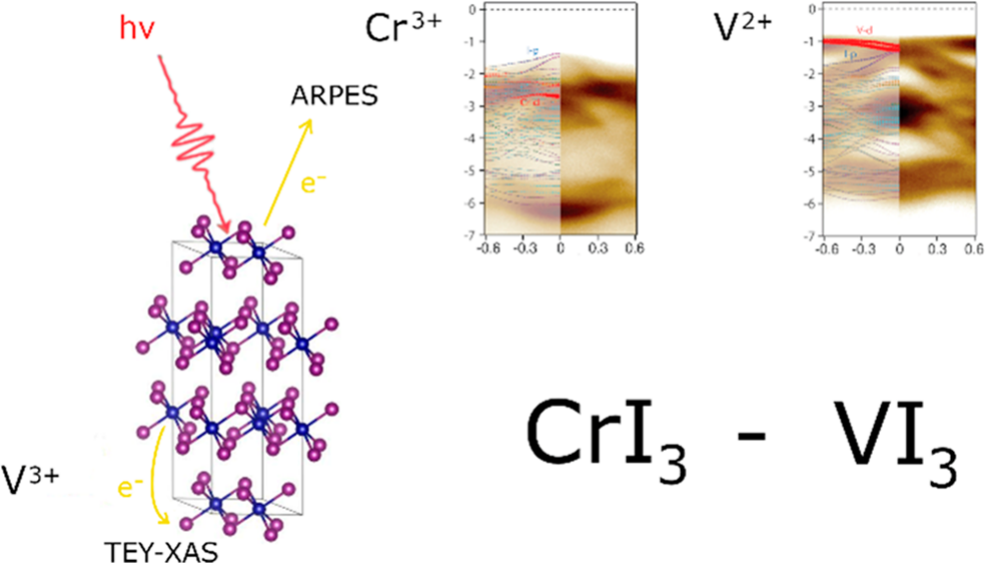

Two-dimensional van der Waals magnetic semiconductors display emergent chemical and physical properties and hold promise for novel optical, electronic and magnetic “few-layers” functionalities. Transition-metal iodides such as CrI_3_ and VI_3_ are relevant for future electronic and spintronic applications; however, detailed experimental information on their ground state electronic properties is lacking often due to their challenging chemical environment. By combining X-ray electron spectroscopies and first-principles calculations, we report a complete determination of CrI_3_ and VI_3_ electronic ground states. We show that the transition metal-induced orbital filling drives the stabilization of distinct electronic phases: a wide bandgap in CrI_3_ and a Mott insulating state in VI_3_. Comparison of surface-sensitive (angular-resolved photoemission spectroscopy) and bulk-sensitive (X-ray absorption spectroscopy) measurements in VI_3_ reveals a surface-only V^2+^ oxidation state, suggesting that ground state electronic properties are strongly influenced by dimensionality effects. Our results have direct implications in band engineering and layer-dependent properties of two-dimensional systems.

Research on two-dimensional (2D) van der Waals (vdW) materials has been recently boosted by the discovery of layer-dependent long-range magnetic order,^1−4^ Dirac physics^5,6^ as well as Mott transitions.^7^ Among 3d transition-metal vdW semiconductors, CrI_3_ and VI_3_ have attracted significant attention, as they undergo structural and electronic transitions as a function of temperature, net long-range magnetization within layers, and in the case of CrI_3_ layered antiferromagnetism.^8−14^ The importance of dimensionality effects has been recognized and for this reason the majority of experimental and theoretical work has focused on monolayers and few-layers films. However, both the interplay of dimensionality with relevant interactions, such as spin–orbit coupling (SOC), and the possible crossover of 3D vs 2D electronic properties are not well understood. In this respect, open questions include: (i) the role of Coulomb interaction and SOC in determining the 3d electronic states and their long- and short-range ordered collective excitations,^15^ (ii) if and how the orbital filling in the electronic ground state is modified at the surface, (iii) what are the changes in the bandwidth and of the hybridization of halogen and transition metal states when dimensionality is reduced. These issues need to be addressed, since they have a significant impact in the potential electronic and spintronic applications, and their control may drive the realization of tailored heterostructures.^16,17^

The crystal structure of MI_3_ (M = Cr, V) is characterized by one M cation surrounded by six I anions, arranged in edge-sharing octahedra. Within the planes, the M atoms are arranged in a honeycomb geometry (Figure 1a). CrI_3_ undergoes a structural transition at *T*_S,CrI3_ = 220 K from the high-temperature monoclinic structure to the low-temperature rhombohedral structure *R*3̅, while VI_3_ changes from the rhombohedral structure *R*3̅ above *T*_S,VI3_ = 79 K to a monoclinic phase below the transition temperature.^18^ Concerning the electronic structure, for VI_3_ two different descriptions were proposed in literature: (i) a metallic ground state, in which the a_1g_ orbital state is fully occupied, and the doubly degenerate e_g_′ orbital state is half occupied;^9,19,20^ (ii) a Mott-insulating ground state, in which the e_g_′ state is fully occupied, while the a_1g_ state is unoccupied.^17,21^ This last picture appears to be consistent with a previous experimental measure of the optical band gap in VI_3_^22^ and a recent spectroscopic investigation;^23^ indeed, no density of states at the Fermi level has been detected. The authors suggest that despite the trigonal distortion in this system being small and thus alone not being able to significantly split the t_2g_ orbital state into e_g_′ and a_1g_ states, the energy gap is opened by the additional on-site Coulomb interaction contribution. However, a firm characterization of the ground state of VI_3_ has not been reached yet.

**Figure 1:**
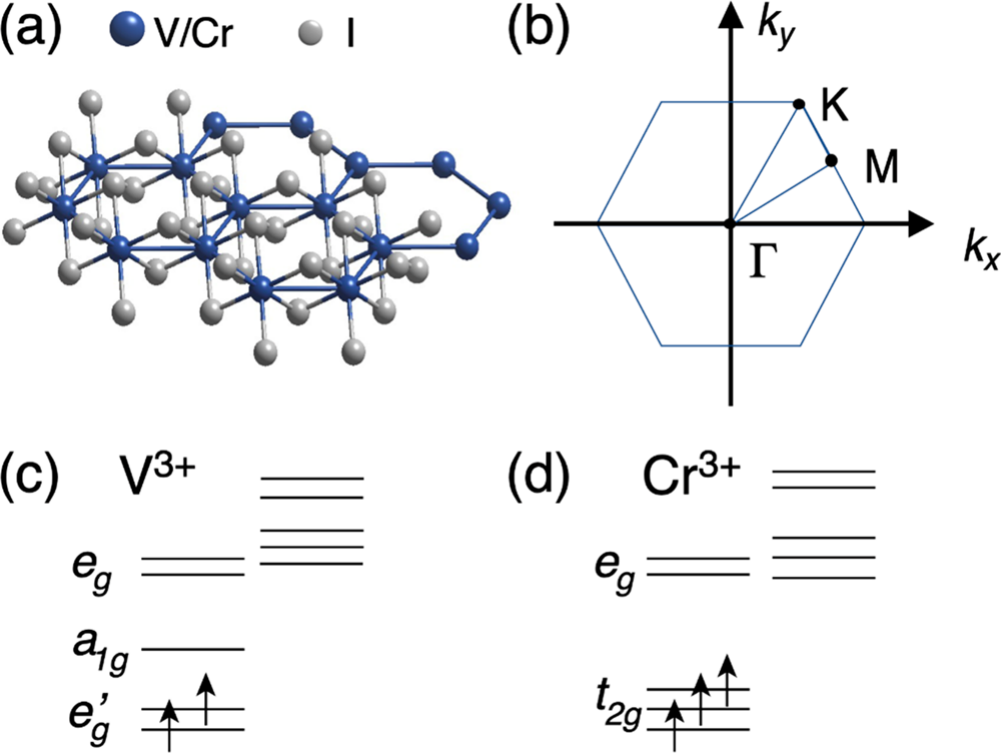
(a) Crystal structure of CrI_3_ and VI_3_ monolayers. (b) Two-dimensional Brillouin zone. Crystal-field splitting and related electron filling for (c) Cr and (d) V.

As for CrI_3_, it has been proposed that a surface structural relaxation may explain the onset of bulk ferromagnetism vs few-layers antiferromagnetism;^24^ it has also been argued that parallel spin states are energetically favored in both stacking configurations, and the result strongly depends on the on-site Coulomb energy employed in the calculations.^17^ Hence, a better understanding of the surface electronic configuration and orbital occupation/arrangement would be helpful to better understand how interlayer interaction affects electronic states.

Motivated by the above issues, in this Letter we report on the determination of the electronic ground state of 3D-crystalline CrI_3_ and VI_3_, by combining different depth-sensitive electron spectroscopies with density functional theory (DFT) calculations for single layers. We observe that in CrI_3_ a wide band gap is opened between majority spin e_g_ and t_2g_ states, whereas in VI_3_ the t_2g_ orbital state is split into (filled) e_g_′ and (empty) a_1g_ orbital states with a narrower band gap between them, consistently with DFT predictions. Surface-sensitive angular-resolved photoemission spectroscopy (ARPES) data reveal the stabilization of a different ground state in VI_3_ at the surface, characterized by a V^2+^ valence and an occupied a_1g_ band, where the bandgap lies between the octahedral-split a_1g_ and the higher-lying empty e_g_ states. In contrast, the 3^+^ oxidation state measured both for bulk CrI_3_ and VI_3_ by X-ray absorption spectroscopy (XAS). Resonant photoemission spectroscopy (ResPES) data, compared to atom- and orbital-resolved density of states obtained from DFT, provide consistent evidence of the orbital character of the different valence band contributions, revealing that the orbital character itself has a profound influence on the ground state electronic properties. The good consistency between experimental data and DFT single-layer calculations suggest that interlayer interactions are less important in determining the stable electronic configuration for both CrI_3_ and VI_3_.

vdW crystals are quasi-two-dimensional systems with little interaction along the *c*-axis layer stacking direction. The extreme surface-sensitivity of ARPES when performed with photon energies in the 20–55 eV range (from 5 to 10 Å,^25^ corresponding to a single layer unit) allows to study the single-layer electronic structure of MI_3_, referring to the surface-projected BZ reported in Figure 1b. We also report, for reference, in Figure 1c,d the expected crystal field splitting and related electronic filling for Cr^3+^ and V^3+^, respectively.

XAS measurements across the V and Cr L_2,3_ edges (Figure 2a,b) confirm the 3^+^ oxidation state of bulk CrI_3_ and VI_3_. As a matter of fact, the line shape and photon energy of L_3_ and L_2_ edges of the former are fully consistent with previous measurements on CrI_3_,^26,27^ while V L_2,3_ edges of the latter closely resemble those of other vanadium compounds with a V^3+^ valence state such as V_2_O_3_, including the characteristic V 2p to V 3d empty t_2g_ transitions in the near-edge regions at 514 and 522 eV.^27,28^ The spectra of both compounds are unaffected by contamination as they were cleaved *in-vacuo*.

**Figure 2:**
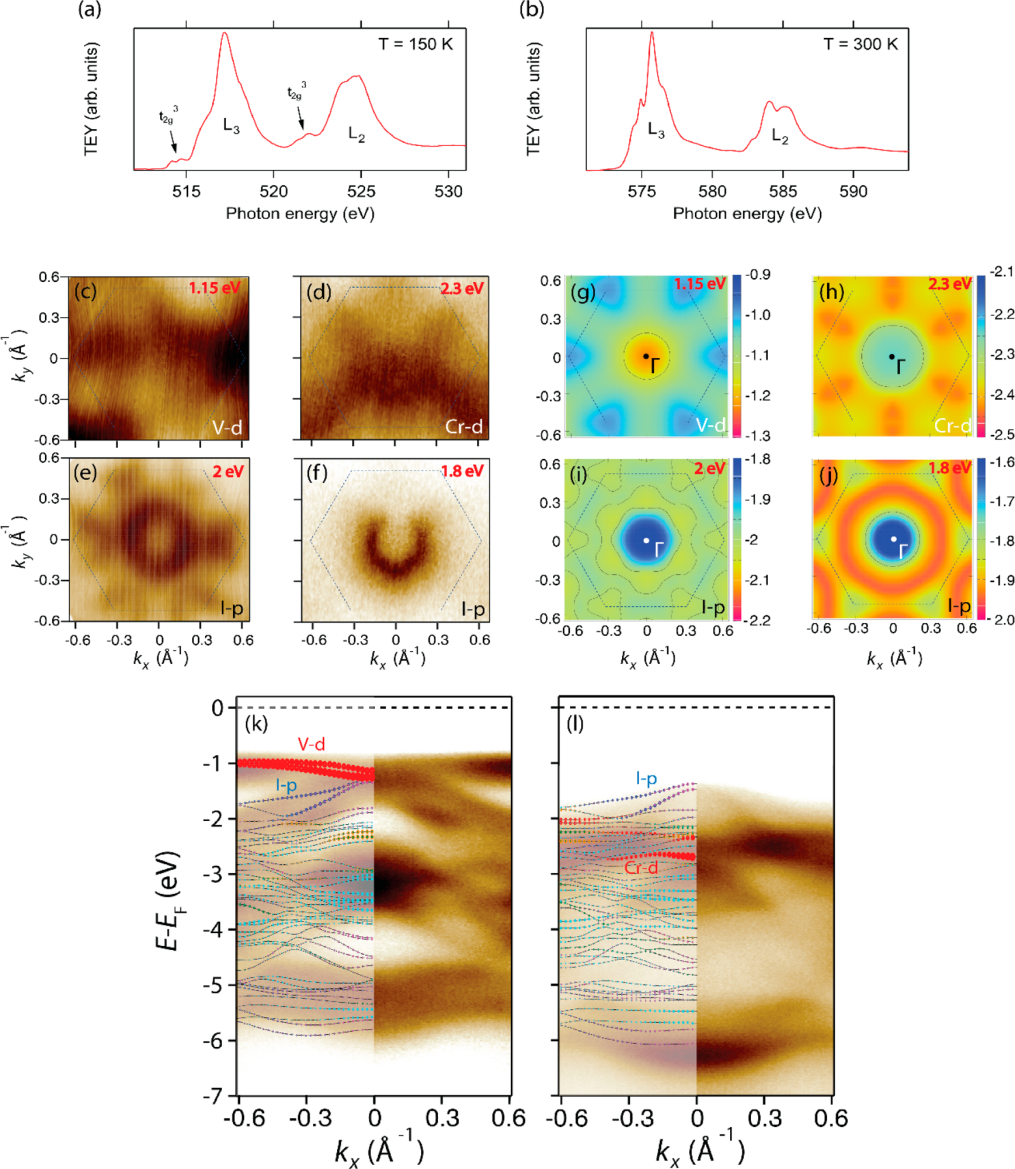
XAS spectrum of CrI_3_ (a) over the Cr L_2,3_ edge (*T* = 300 K), and VI_3_ (b) over the V L_2,3_ edge (*T* = 150 K). ARPES isoenergetic *k*_*x*_–*k*_*y*_ maps of VI_3_ (*T* = 150 K, *hv* = 32 eV) at (c) 1.15 eV and (e) 2 eV, and CrI_3_ (*T* = 300 K, *hv* = 41 eV) at (d) 2.3 eV and (f) 1.8 eV, highlight the 3-fold symmetry of V/Cr-d states and the 6-fold symmetry of I-p states. The energy in red in the top-right corner of each map pinpoints the binding energy of the isoenergetic cut. DFT calculation of the total energy map with contours shown as black lines for specific energies of VI_3_, (g) 1.15 eV, (i) 2 eV and CrI_3_, (h) 2.3 eV, (j) 1.8 eV. Dashed lines in each image represent the 2D hexagonal first Brillouin zone. The energy in red in the top right corner of each map is the binding energy of the isoenergetic cut. ARPES spectra of (k) VI_3_ (*T* = 150 K, *hv* = 32 eV) and (l) CrI_3_ (*T* = 300 K, *hv* = 32 eV) along the Γ–K direction. Theoretical band structures with FM configuration and SOC for VI_3_ (GGA+U, U = 2 eV) and CrI_3_ (bare GGA) monolayers are superimposed on experimental data. The colors highlighting the bands represent the following components for V/Cr d-orbital, red denotes d_3*z*2–*r*2_, green denotes d_*xz*_ and d_*yz*_, yellow denotes d_*xy*_ and d_*x*_2_–*y*_2__. For I p-orbital, gray denotes p_*z*_, magenta denotes p_*y*_, blue denotes p_*z*_.

XAS spectra are intrinsically integrated over a thickness of 4–6 nm of material,^29^ and as such it does not retain information coming from the very first layer of CrI_3_ and VI_3_. The direct surface analysis and investigation of the energy and the overall symmetry of the electronic states of VI_3_ and CrI_3_ was addressed by collecting *k*_*x*_–*k*_y_ photoemission intensity maps at constant energy (see Figure 2c–f) and energy-momentum spectra, (see Figure 2k–l). Experimental results were compared to DFT calculated electronic structures, where the U value was changed in the range between 0 and 3 eV (see SI, Methods and Figure S1) for both materials, seeking the best agreement with ARPES data. The calculations were performed for a single-layer with FM configuration, i.e. the magnetic ground state in monolayer form for VI_3_ and CrI_3_, both with and without SOC. The magnetization direction, when SOC was included, was out-of-plane, consistently with the orientation of the magnetic moments. We note that our ARPES data were measured above the Curie temperature; nevertheless, a much better agreement is found with spin-polarized DFT, rather than with nonmagnetic DFT calculations. The latter would in fact result in a metallic ground state, inconsistent with experimental results on these materials. Local magnetic correlations may actually occur even above the Curie temperature and this is implied by the good agreement of experimental data and spin-polarized DFT, even in the absence of long-range magnetic ordering.

Our experimental data show the existence of a sizable bandgap (the distance between the valence band maximum and the conduction band minimum) for both systems and allow us to evaluate the bandgap energies to be larger than 0.9 eV for VI_3_ and 1.35 eV for CrI_3_. These values have been obtained by comparing ARPES data to the experimentally determined Fermi level (see Methods). It is important to underline that the experimental bandgap values are lower limits for the full bandgap, as ARPES detects occupied electron-density of states (DOS) only; in addition, the presence of small charging effects of cleaved surfaces^30−32^ cannot be excluded. The latter are common and well-understood in ARPES measurements of insulating compounds and can determine an artifactual rigid shift of the Fermi level of a few hundreds of millielectronvolts, without further changes in the band structure, as we verified in our measurements (see SI Figure S2). We note that we cannot use DFT calculations (as reported in SI Figure S3) for quantitative comparison, as the underestimate of band gaps is a well-known DFT problem in treating excited states.^33^ Nevertheless, a good agreement with previous experimental results is found, yielding a similar value obtained from optical measurements for CrI_3_^34^ and also both from optical^12,22^ and spectroscopic^23^ measurements for VI_3_.

From Figure 2, we note that the overall electronic structures of VI_3_ and CrI_3_ show several similarities. In fact, the 3*d* electronic states originating from V or Cr give rise to similar nearly dispersionless features in the VB, whose intensity is prominent in the collected spectra (Figure 2k,l). On the other hand, the I-derived 5p orbitals are very dispersive and the orbital-mixing is strong for both VI_3_ and CrI_3_. The orbital character of these bands, as inferred from DFT, shows that the SOC is crucial for explaining the observed energy-momentum spectra. The effect of SOC modulates the band structure involving the V/Cr d_*z*2–3*r*2_ and I p_*x*_–p_*y*_ orbitals at binding energies of ∼1 eV in VI_3_ and around 2.5 eV in CrI_3_. Moreover, the inclusion of SOC is relevant in closing the gap between V a_1g_ and I p bands at Γ (see SI Figure S1).

Despite I p bands being fairly similar, our results show that VI_3_ and CrI_3_ behave in a different way from the electronic point of view. To emphasize this point, we carried out polarization-dependent measurements on both compounds. p-polarized light has both in-plane and out-of-plane components of the wavevector with respect to the sample surface, whereas s-polarized light has only the in-plane component. By exploiting the light polarization dependence we are thus selectively sensitive to in- and out-of- plane orbitals (Figure 3). For VI_3_, we clearly see in Figure 3a that the orbitals contributing to the spectroscopic signal at ∼1 eV are mostly out of plane, that is, those with a_1g_ character. A value of U = 2 eV is thus found to best simulate our data. Higher values of U would shift the V a_1g_ states up toward the Fermi level, opening a gap between them and the I bands which is not observed in the experiment (Figure S1h). Lower values of U, instead, would place V d_*xz*_ and d_*yz*_ orbitals at an energy of ∼1 eV (Figure S1e-f) which is inconsistent with the data.

**Figure 3:**
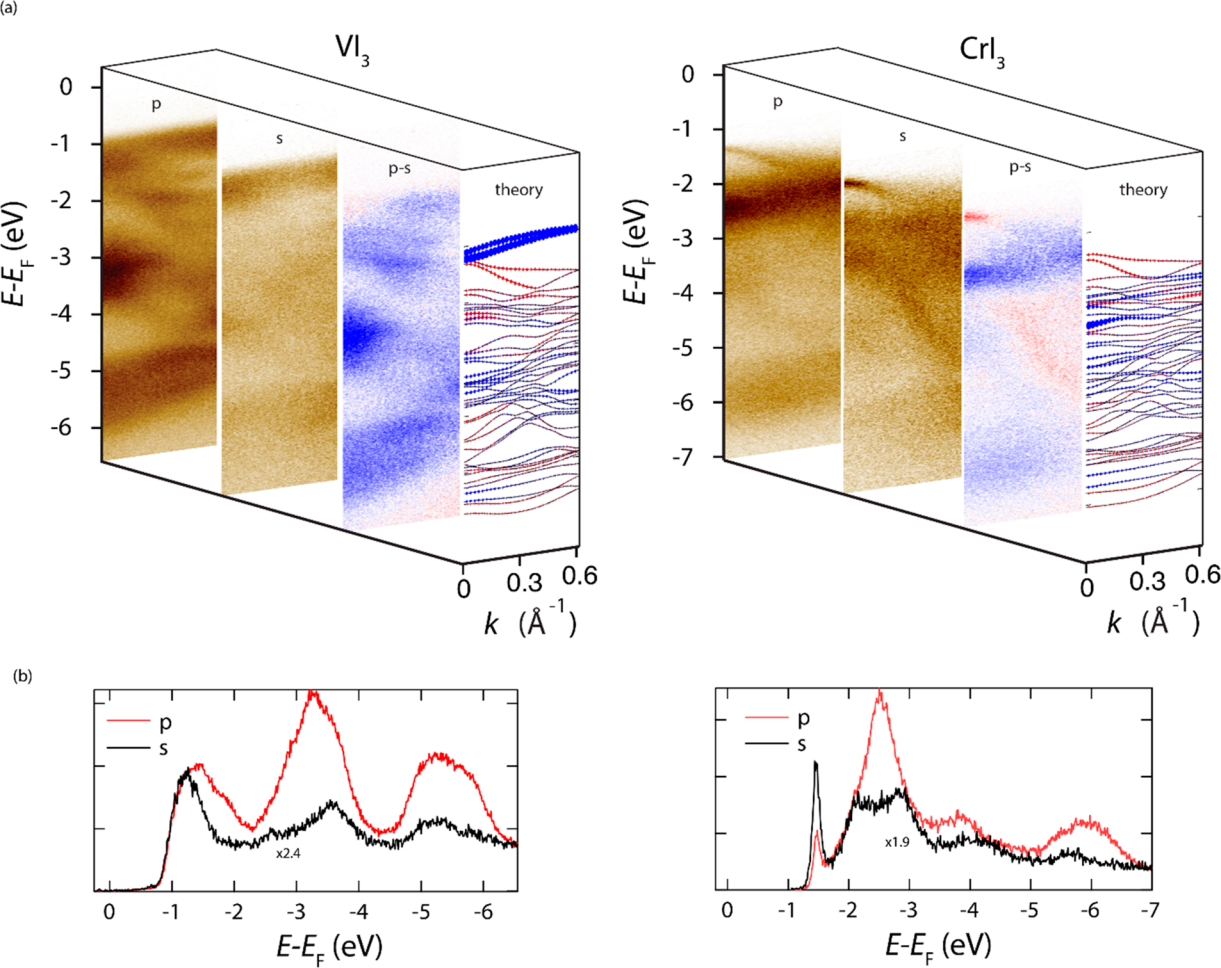
(a) VI_3_ (left) and CrI_3_ (right) spectra as a function of light polarization (p-polarization, s-polarization, difference p–s). p-polarized light has an out-of-plane component, while s-polarized light is completely in-plane. The “theory” panel displays the DFT band structure; a blue color indicates bands with out-of-plane component, while a red color emphasizes in-plane bands. (b) Angle-integrated spectra of VI_3_ (left) and CrI_3_ (right) evidencing polarization-dependent intensity of specific band features.

ARPES results for CrI_3_ are in striking difference with those shown above for VI_3_. First, from Figure 3a we deduce that bands at 2.5 eV BE are ascribed to the out-of-plane d_3z2-r2_ orbitals. Second, we observe that, as soon as U values different from zero are introduced in the DFT calculations, those orbitals move to higher BEs (Figure S1n–p), a feature that is not observed by ARPES. This can be interpreted as follows. CrI_3_ exhibits t_2g_–e_g_ crystal-field splitting of d states; the different orbital filling of Cr^3+^ ions compared to V^3+^ results in a t_2g_ level completely filled by majority electrons. The orbital splitting between e_g_ and t_2g_ orbital states is sufficiently large in this case to stabilize a fully insulating state, even in the absence of a finite U-value within the DFT+U approach.

Another relevant difference between the two compounds is that DFT calculations compare well with ARPES spectra with the exception of the a_1g_ orbital filling of VI_3_. The a_1g_ are clearly revealed as filled states by ARPES, while are predicted to be empty by DFT consistently with a V^3+^ ionic charge. On top of this, XAS results also indicate a V^3+^ valency of the bulk. This peculiar behavior is nevertheless clearly revealed by ARPES, which probes just the topmost layer of the material, therefore including intrinsic and extrinsic surface effects like relaxation and defect-doping effects that can be responsible of the local electron filling of the a_1g_ band. We thus suggest that a different ground state, characterized by a V^2+^ orbital filling, stabilizes at the surface: it turns out that the gap between filled and empty states observed in ARPES measurements is opened by the octahedral crystal field, rather than that by the bulk trigonal crystal field splitting of a_1g_ and e_g_′ levels. On the other hand, CrI_3_ does not show any evidence of a different surface electronic environment.

In Figure 2c–f, we note that the constant energy ARPES maps for VI_3_ and CrI_3_ display a different symmetry in connection with the orbital character of the electronic states. I 5p states display an apparent 6-fold symmetry, while V and Cr 3d states show a 3-fold symmetric pattern, differently from DFT results (reported in Figure 2g–j). The 3-fold symmetry of V 3d states is highlighted also in SI Figure S4. Indeed, the expected trigonal symmetry is not necessarily reproduced in our single-layer calculations, since the primitive cell encompasses two layers. In previous works, it has been suggested that such a pattern could be connected with the onset of the ferromagnetic ground state,^23^ that breaks time-reversal symmetry when VI_3_ undergoes the magnetic transition. At the surface, this combines with the loss of inversion symmetry, giving rise to a *P–T* symmetry-breaking system. Our ARPES data (150 K for VI_3_, 300 K for CrI_3_) were collected at sample temperatures well above the Curie point (CrI_3_, *T*_C,CrI3_ = 61 K; VI_3_, *T*_C,VI3_ = 50 K), and suggest a different interpretation with respect to the one given in ref (23). The reduction of symmetry of ARPES constant energy maps with respect to DFT results may be ascribed to surface effects, that are not accounted for in the DFT simulations of VI_3_ and CrI_3_ single-layers (i.e., not for semi-infinite crystals). However, we cannot rule out the existence of magnetic fluctuations and their role in breaking time-reversal symmetry.

The relevance of short-range magnetic interactions in vdW magnetic materials, including VI_3_, has been pointed out by numerous studies.^13,35−38^ Well above the Curie temperature, thermal fluctuations randomly orient the electron spins in the valence band, but the nonmagnetic ground state can be locally described in terms of orbital filling of the TM states by majority electrons. The occurrence of magnetic fluctuations well above the Curie point in the absence of long-range order is well-documented for correlated materials;^39−42^ the observed narrow bandwidth of V- and Cr-derived 3d bands hints at the importance of electron–electron interactions as well. Furthermore, V- and Cr-projected states would be more affected by short-range correlation effects, while the I contribution to the magnetic moment is negligible,^9^ leaving the symmetry of I 5p states as dictated by the structure. The intralayer atomic arrangement does not change even at the structural transition that both crystals undergo at *T*_S,CrI3_ = 220 K and *T*_S,VI3_ = 78 K, involving only layer stacking. The comparison of calculations and surface-sensitive experimental data allows us to conclude that the band structure is largely unaffected across the structural transitions.

We also measured ResPES, exploiting the selective abrupt changes of photoionization cross sections, to identify the orbital contributions to the spectra. V 3d orbitals contributing to the bands at 1 eV BE, and similarly Cr 3d orbitals contributing to the bands at 2.5 eV BE, are resonantly enhanced when the photon energy reaches the photoionization threshold of the V and Cr 3p core levels, respectively. In this way, we experimentally probe the atomic character of the wave functions contributing to ARPES intensities. The large signal enhancement of the nondispersing bands when the photon energy coincides with the 3p edges (Figure 4a,c) is a direct signature of their V/Cr orbital nature, and can be mapped onto the DFT orbital projection of the DOS (Figure 4b,d).

**Figure 4:**
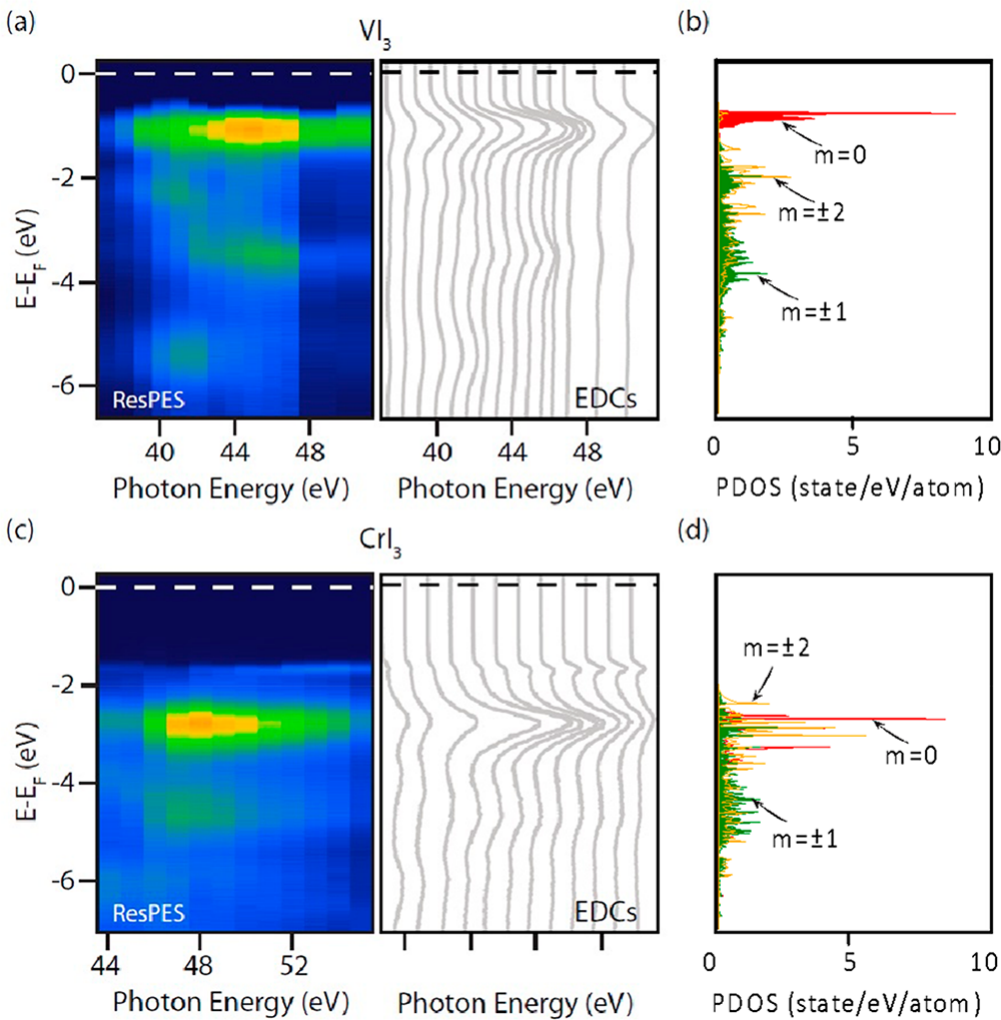
(a) ResPES in the first BZ for VI_3_: the color map (left panels) displays the momentum-integrated photoemission intensity; the resonant EDCs (right panels) emphasize the band dispersion (or lack of) along the measured photon energy. (b) d*-*orbital projection DOS of VI_3_; the color scale represents d orbital characters as follows: filled red, m = 0; filled green, m = 1; orange, m = 2. (c,d) Same as (a,b), but for CrI_3_.

Photon energy dependent ARPES, such as ResPES, intrinsically also probes the k_*z*_ dispersion. The absence of dispersion of both V and Cr projected bands, highlighted by the EDC spectra (Figure 4a–c, right panel), indicates that those states are “2D-like” with negligible interlayer interaction (assuming no artifacts are introduced by rescaling the spectra to compensate charging effects). The consistency of ARPES results with single-layer calculations appears therefore strengthened.

In summary, we characterized the ground-state electronic structure of CrI_3_ and VI_3_: based on the excellent agreement between photoemission data (angle-resolved, light polarization-dependent and resonant) and DFT-calculated band dispersion and orbital-resolved density of states, we give evidence of how substantially different can be the orbital configuration of magnetic trihalides for different TM ions. CrI_3_ shows a 3-fold t_2g_ orbital degeneracy and a wide bandgap, while VI_3_ shows a Mott-insulator-like ground state with a_1g_-e_g_′ orbital splitting and a narrower bandgap. Single-layer band structure calculations compare well with ARPES spectra suggesting weak electronic interaction between layers, including between the surface layer and the bulk. Moreover, the occupancy of the a_1g_ state at the surface of VI_3_ indicates that its surface is stabilized by an unconventional V^2+^ state, at variance with the V^3+^ bulk valency. Therefore, future research in ultrathin films or nanoparticles of MI_3_ van der Waals materials with variable surface to bulk ratios should carefully explore the effective ionic configurations that determine their electronic properties and related potential functionalities.

## Supporting Information Available

Methods and experimental details, beamline specifications, additional calculations (PDF)
