# Supplementary material for: Influence of Orbital Character on the Ground State Electronic Properties in the van Der Waals Transition Metal Iodides VI3 and CrI3
Source: Nano Lett. 2022 Aug 30;22(17):7034–41. doi: 10.1021/acs.nanolett.2c01922 (PMC9479147; doi:10.1021/acs.nanolett.2c01922)
Supplement: Supplementary file 1 — nl2c01922_si_001.pdf [file nl2c01922_si_001.pdf]

Influence of orbital character on the ground state electronic properties in the van der Waals transition-metal-iodides VI_3 and CrI_3

*Alessandro De Vita[1,2‡], Thao Thi Phuong Nguyen[3,4‡], Roberto Sant[5], Gian Marco Pierantozzi[1], Danila Amoroso[6,7], Chiara Bigi[1,8], Vincent Polewczyk[1], Giovanni Vinai[1], Loi T. Nguyen[9], Tai Kong[9], Jun Fujii[1], Ivana Vobornik[1], Nicholas B. Brookes[5], Giorgio Rossi[1,2], Robert J. Cava[9], Federico Mazzola[1], Kunihiko Yamauchi[3,4], Silvia Picozzi[6], and Giancarlo Panaccione[1]**

[1]Istituto Officina dei Materiali (IOM)-CNR, Laboratorio TASC, in Area Science Park, S.S.14, Km 163.5, I-34149 Trieste, Italy.

[2]Dipartimento di Fisica, Università di Milano, Via Celoria 16, I-20133 Milano, Italy

[3]Institute of Scientific and Industrial Research, Osaka University, 8-1 Mihogaoka Ibaraki, Osaka 567-0047, Japan

[4]Department of Precision Engineering, Graduate School of Engineering, Osaka University, 2-1 Yamadaoka, Suita, Osaka 565-0871, Japan

[5]ESRF, The European Synchrotron, 71Avenue des Martyrs, F-38043 Grenoble, France

[6]Consiglio Nazionale delle Ricerche (CNR-SPIN), Unità di Ricerca presso Terzi c/o Università "G. D'Annunzio", 66100 Chieti, Italy

[7]NanoMat/Q-mat/CESAM,Université de Liège, B−4000 Liege, Belgium

[8]School of Physics and Astronomy, University of St Andrews, St Andrews KY16 9SS, United Kingdom

[9]Department of Chemistry, Princeton University, Princeton, NJ, 08540, USA.

‡ These authors contributed equally to this work.

* Corresponding author: G. Panaccione

Email: panaccione@iom.cnr.it

Supplementary:

Methods

ARPES: Commercially available VI_3 and CrI_3 crystals were mounted in an N_2 glovebox environment; in order not to expose them to air, they were transferred in N_2 atmosphere to the ultrahigh-vacuum (UHV) chamber system by means of a specifically designed suitcase, prior to be cleaved at p= $5x10^{-10}$ mbar. The samples were measured at the APE-LE beamline of the synchrotron radiation source Elettra (Trieste, Italy) [41] by using a Scienta DA30 hemispherical analyzer. The ARPES experiments were performed at a base pressure better than $5x10^{-10}$ mbar using p-polarized light (45° incidence, normal emission), unless otherwise specified. Samples were grounded and measurements of the Fermi edge from a polycrystalline Au foil have been used to estimate the position of the Fermi level and help in compensating charging effects, as explained in the eponymous section.

The sample is oriented such that the analyzer slit, and thus the probed momentum, lies along the Γ-K symmetry direction. In our experimental geometry, s-polarized photons have only an in-plane component parallel to the analyzer slit and hence in the mirror plane; p-polarized photons instead have an in-plane orthogonal to the analyzer slit and hence to the mirror plane, but they also possess an out-of-plane component which by definition lies in the mirror plane. This implies that both light polarization vectors have one component (in-plane for s-polarization, out-of-plane for p-polarization) which is symmetric with respect to the mirror plane, so that non-zero matrix elements are always expected.

XAS: XAS measurements have been performed at the APE-HE beamline of the synchrotron radiation source Elettra (Trieste, Italy) [41], in total electron yield (TEY) mode, at a base pressure better than $5x10^{-10}$ mbar. p-polarized light was used, with the sample surface placed at 45° with respect to the beam.

DFT: First-principles calculations based on density-functional theory (DFT) were performed using the VASP code [42] within the generalized gradient approximation (GGA) [43]. Within a DFT+U approach, when considering correlation effects of transition-metal 3*d* orbitals [17], we set the on-site effective Hubbard interaction in the range from 0 eV to 3 eV. The experimental values were employed for the in-plane lattice constants of VI_3 as a = 6.84 Å [13] and CrI_3 as a = 6.87 Å [14]. The monolayer was placed under a vacuum layer of 20 Å. After the atomic structure was optimized using a *k*-point grid of 6×6×1 until forces acting on atoms were smaller than 0.0001 eV/Å, their electronic properties were calculated by using 12×12×1 *k*-points mesh. The spin-orbit interaction was included self-consistently in the calculation.

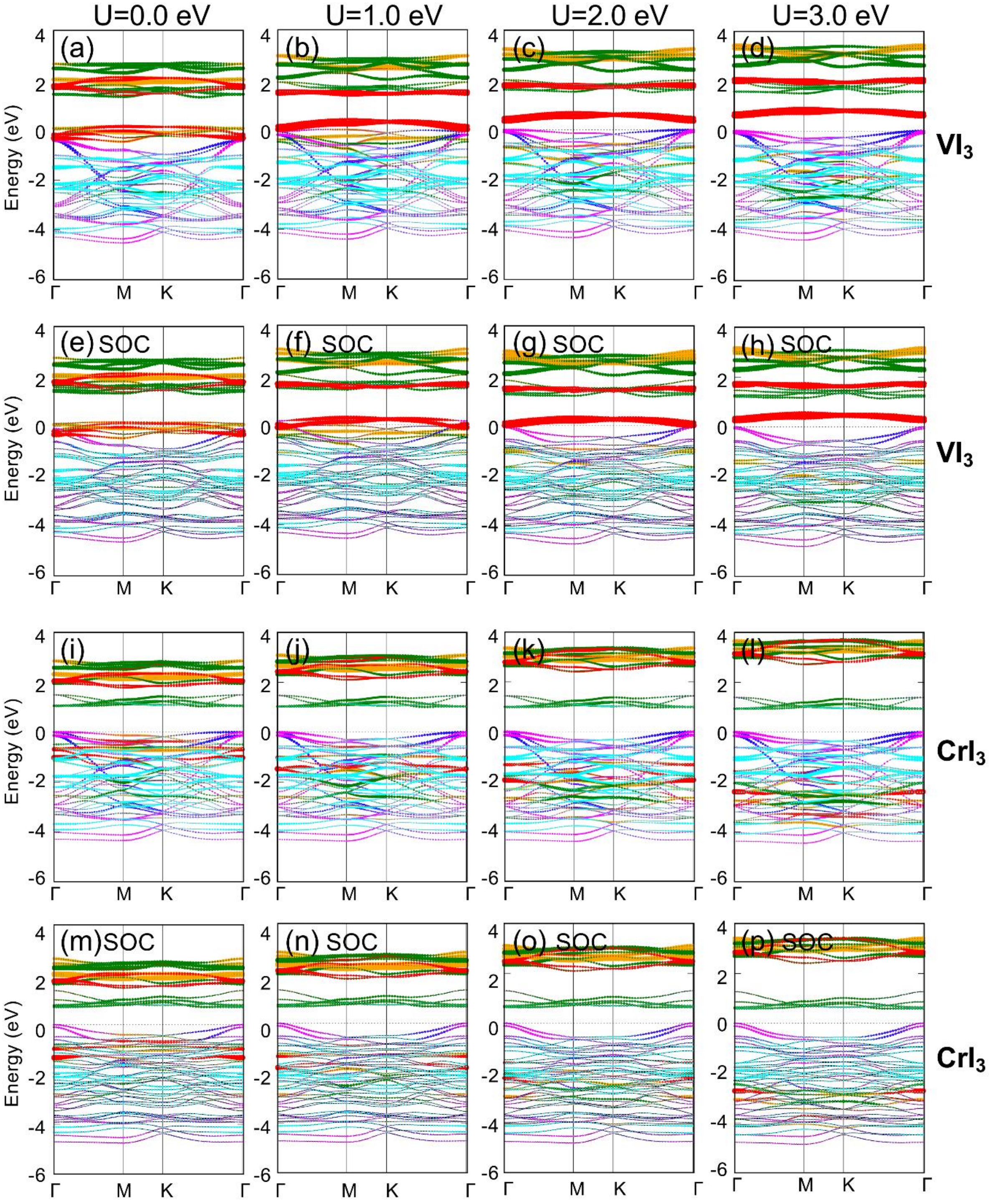

Figure S1: Calculated band structures within GGA and Coulomb interaction Hubbard U values from 0 eV to 3 eV; (a-d) without SOC and (e-h) with SOC for VI_3 monolayer. The color highlighting the bands represent components as follows: V/Cr *d*-orbital; red: d_{3z2-r2}, green: d_{xz} and d_{yz}, yellow: d_{xy} and d_{x2-y2}, and I *p*-orbital; grey: p_x, magenta: p_y, blue: p_z. The Fermi energy is set at zero. (i-p) same with (a-h) but for CrI_3 monolayer.

Charging effects – Due to the sizeable bandgap, samples exhibit charging upon photon flux exposure. Measurements have been performed with decreasing photon flux, in order to assess the charging effect on the band structure (Fig. S2): the resulting spectra show that, if the sample temperature is not too low, charging does not influence the lineshape but rigidly shifts the curve towards higher BEs.

We set the minimum temperature for our measurement by noticing when charging effects start becoming more problematic, i.e. when spectral deformation takes place. Measuring at any lower temperature than 150 K for VI_3 and 300 K for CrI_3 foils any attempt of reproducibility in our case.

Since sample charging only affects the position of the spectrum in kinetic energy and not its lineshape, this affects the estimation of the position of the Fermi level. We reduced the photon fluence of the measurement until no shift is observed, as shown in Fig. S2. We set this experimental condition as representative of a non-charging spectrum.

At the same time, we acquired the spectrum from a polycrystalline Au foil across the Fermi edge: the correspondence between these two independent measurements allowed us to properly rescale the kinetic energy scale to binding energy on the non-charging spectrum. This acted as a reference for the evaluation of any charging effect shift of the other spectra.

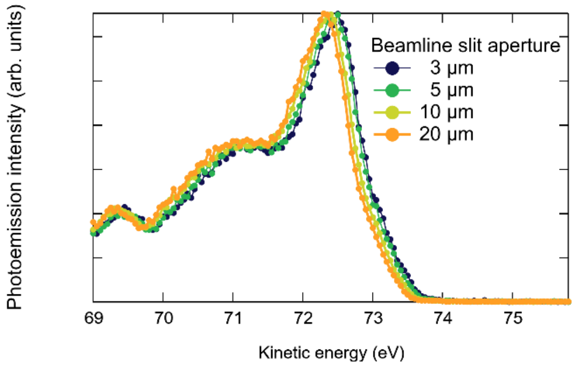

Fig. S2: Angle-integrated spectra of CrI_3 (hv = 80 eV, T = 300 K) as a function of the aperture of the beamline slit, regulating the photon flux impinging on the sample. Charging effects manifest at higher fluxes with a rigid shift of the spectrum towards lower kinetic energies.

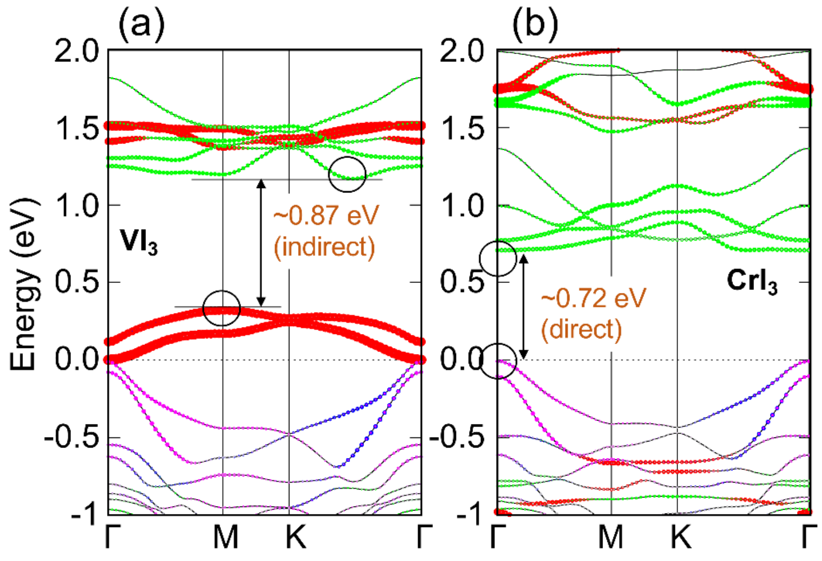

Figure S3: Calculated band structure with SOC taken into account for (a) monolayer VI_3, (GGA+U, U=2eV) with a_{1g}-e_g indirect energy gap ~0.87 eV and (b) monolayer CrI_3, (GGA) with direct band gap ~0.72 eV. The color highlighting the bands represent components as follows: V/Cr *d*-orbital; red: d_{3z2-r2}, green: d_{xz} and d_{yz}, yellow: d_{xy} and d_{x2-y2}, and I *p*-orbital; grey: p_z, magenta: p_y, blue: p_z.

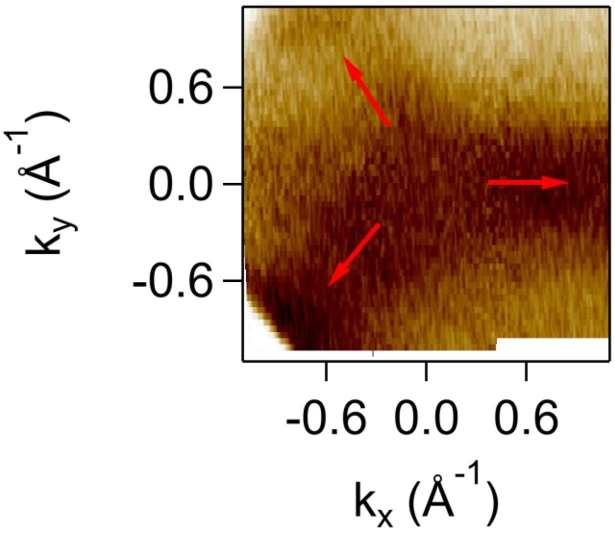

Figure S4: ARPES isoenergetic k_x-k_y map on VI_3 at (hv = 80 eV, T = 150 K) at 1.15 eV BE. The red arrows highlight the threefold symmetry of the V band.

k_z dispersion - The claim that electronic states in VI_3 and CrI_3 mostly show a two-dimensional nature can be further supported by directly showing the k_z band dispersion. Figure S5 exemplifies this point for VI_3: bands are mostly flat across the probed k_z range. We chose to display the cut at $k_x = 0.33$ Å^{-1} in order to clearly separate the two I-derived bands around 2 eV BE: notably, we see how the topmost band is slightly dispersing in k_z, whereas the lower one is totally flat. Across this photon energy interval, almost the entirety of the Brillouin zone is

scanned (Fig. S5b). Since crystalline parameters are quite similar for CrI_3, in light of the lack of dispersion of CrI_3 bands shown in ResPES data in Fig. 4c of the main text we consider this conclusion valid for CrI_3 as well.

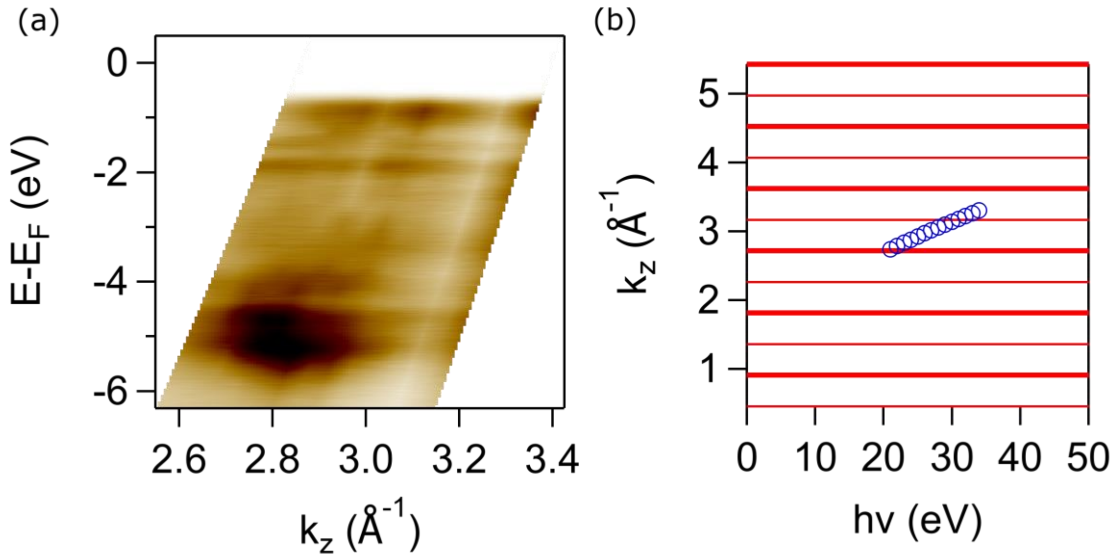

Figure S5: (a) k_z dispersion of the valence band structure of VI_3 (k_x = 0.33 Å^{-1}, in order to clearly discern the I-derived bands around 2 eV BE). (b) Calculated k_z as a function of the photon energy scanned in our experiments on VI_3. Thick red lines represent the borders of Brillouin zones along k_z. We considered an inner potential of V_0 = 15 eV.
